# Programmes of Medical Societies

**Published:** 1962

**Authors:** 


### *Programmes of Medical Societies*

### BRISTOL MEDICO-CHIRURGICAL SOCIETY

*Hon. Sec.:* Dr. A. T. M. Roberts, 12 Southfield Road, Westbury-on-Trym, Bristol.

10th October: **Annual General Meeting** and **Presidential Address.**
November: No Meeting. B.M.A. Televised Clinical Demonstration.
12th December: Dr. R. F. Barbour.
9th January: Dr. J. E. Cates.
13th February: Mr. P. Jardine.
13th March: **Clinical Meeting** at Southmead Hospital.
10th April: Lord Justice Donovan (President's Guest).
8th May: Mr. Rodney Maingot (Society's Guest).

### B.M.A. (South Western Branch)

*Hon. Sec.:* Dr. F. E. Graham-Bonnalie, 37 The Strand, Topsham, nr. Exeter.

30th Sept.: **Meeting** of Presidents, Chairmen and Hon. Secs. of Divisions at Manor House Hotel, Moretonhampstead.
27th Oct.: **Meeting** at Plymouth.

### B.M.A. (Exeter Division)

*Hon. Sec.:* Dr. F. E. Graham-Bonnalie, 37 The Strand, Topsham, nr. Exeter.

18th Oct.: **Meeting** at Royal Devon & Exeter Hospital. Speaker, Miss Honor Wyatt.
29th Nov.: **Combined Meeting** with the Legal Society at the Royal Devon & Exeter Hospital.

### CORNWALL CLINICAL SOCIETY

*Hon. Sec.:* Dr. B. A. Gwynne Jenkins, Dept. of Chest Diseases, 1 Penlu Terrace, Tuckingmill, Camborne, Cornwall

4th Oct.: **Presidential Address on "Anaemia".** R.C.I.
3rd Nov.: **"Emergencies: Modern Anaesthesia"**, by Dr. Trotter, Redruth.
7th Dec.: **Ladies' Night.** Dr. H. P. Speed on **"Poltergeists".** R.C.I.
4th Jan.: **Clinical Meeting.** Redruth.
1st Feb.: **"Pain of Arterial Origin"**, by Mr. H. H. G. Eastcott. R.C.I.
7th Mar.: **Clinical Evening.** St. Lawrence's.
4th Apr.: **B.M.A. Lecture.** R.C.I.
2nd May: **A.G.M.** and **Brains Trust.** Redruth.

### ROYAL DEVON & EXETER HOSPITAL POSTGRADUATE COURSE

*Hon. Sec.:* Mr. H. Dendy Moore, 4 Magdalen Road, Exeter.

9th Oct.: **"Physical Signs in Neurology"**, by Dr. N. S. Alcock.
16th Oct.: **"Modern Concept of the Acute Abdomen"**, by Mr. K. D. J. Vowles.
23rd Oct.: **"Hypertension and Coronary Disease"**, by Dr. G. R. Steed.
30th Oct.: **"The Causes of Debility and/or Loss of Weight"**, by Dr. C. M. Seward.
6th Nov.: **"Present Day Trends in Immunization"**, by Dr. Brendan Moore.
13th Nov.: **"Some Recent Advances in the Treatment of Skin Diseases"**, by Dr. J. R. Simpson.
20th Nov.: **"The Indications for and the Management of Ileostomies and Colostomies"**, by Mr. C. P. Sames.
27th Nov.: **"The Treatment of Cardiac Arrest"**, by Dr. K. G. Lupprian.
4th Dec.: **"Diagnosis and Treatment of Congenital Abnormalities of the Gut in Infants"**, by Mr. J. P. Partridge.

### WEST OF ENGLAND CHILD HEALTH GROUP

*Chairman:* Miss L. M. Bendall, Chief Nursing Officer, City & County of Bristol
*Hon. Sec.:* Dr. A. L. Smallwood, Dept. of Public Health, Tower Hill, Bristol 2

2nd Oct., at 8.15 p.m.: **"The Prevention of Congenital Abnormalities"**, by Dr. R. W. Smithells, Lecturer in Child Health, University of Liverpool.

7th Nov., at 5.30 p.m.: **"The Duke of Edinburgh's Award Scheme"**, by Dr. Allan F. Rogers, Senior Lecturer in Physiology, University of Bristol.

6th Dec., at 8.15 p.m.: **"Parent Counselling"**, by Professor A. G. Watkins, Professor of Child Health, University of Wales.

16th Jan., at 5.30 p.m.: **"Some Problems of the Care and Protection Court"**, by Mrs. Nancy Burton, Chairman of the Magistrates, Juvenile Court, Bristol.

7th Feb., at 8.15 p.m.: **"The Work of the Home and Hospital Teacher"**, by Mr. C. Williams, Headmaster, South Bristol School, Mr. C. Meese, Hospital Teacher, and Miss D. Watkins, Home Teacher.

6th Mar., at 5.30 p.m.: **"Teds, Teens and Trads"**, by the Rev. E. C. Marvin, Minister, St. James's Presbyterian Church, Lockleaze.

11th May: **Clinical Meeting** at Hortham Hospital, Almondsbury.

Meetings take place in the Lecture Theatre at the Children's Hospital, unless otherwise stated.

### B.M.A. (Bristol Division)

*Hon. Sec.:* Dr. H. Temple Phillips, Berkeley House, Charlton Road, Westbury-on-Trym, Bristol

17th Oct., at 8.30 p.m.: **Annual General Meeting** at the Royal Fort.

21st Oct., at 2.45 p.m.: **St. Luke's Tide Service** at Bristol Cathedral. Preacher, the Right Rev. the Bishop of Sheffield.

6th Nov., at 8.0 p.m.: **Annual Dance** at the Berkeley Café.

14th, 15th and 16th Nov., at 8.0 p.m.: **Colour Television Demonstrations of Clinical Subjects.** By arrangement with Smith, Kline & French Laboratories Ltd. At the Royal Fort.

16th Jan., at 8.30 p.m.: **Lecture** by Professor C. F. Powell, M.A., F.R.S. Preceded by **Dinner** in the Senior Common Room. At the Royal Fort.

20th Feb., at 8.30 p.m.: **Lecture** by Miss E. Ralph, City Archivist on **"Famous Bristol Women Through the Ages".**

20th Mar., at 8.30 p.m.: **Lecture** by Macdonald Critchley, M.D., F.R.C.P. Preceded by **Dinner** in the Senior Common Room. At the Royal Fort.

### B.M.A. (Gloucestershire Branch)

*Hon. Sec.:* Dr. H. G. Scott-Kerr, 29 Leckhampton Road, Cheltenham

11th Oct.: **Presidential Address: "Leg-pulling and Other Exercises in General Practice."** At Cheltenham.

8th Nov.: **Annual Report of Branch Council & Financial Statement. Representatives' Report.** Paper: **"Sudden Blindness"** Illustrated. Mr. Douglas S. Thomson. At Gloucester.

13th Dec.: Address: **"A few Diseases which are of common interest to the Medical and Veterinary Professions".** Sir John N. Ritchie, C.B., Chief Veterinary Officer, Ministry of Agriculture Fisheries and Food.

A joint Meeting with the Members of the "Cotswold Clinical Club of Veterinary Surgeons" At Cheltenham.

3rd Jan.: **Dinner Dance.** At the Chase Hotel, nr. Ross-on-Wye.

10th Jan., at 8.0 p.m.: Conjoint Address: **"The Anaemias of Pregnancy".** Illustrated. Mr. E. M. Edwards and Dr. A. B. Hill. At Gloucester.

14th Feb., at 5.30 p.m.: **Clinical Meeting.** At Cheltenham.

14th Mar.: Paper: **"Fibrinolysis and Occlusive Vascular Disease".** Dr. G. R. Fearnley. At Gloucester.

11th Apr.: Address: **"Television and Medicine".** B.M.A. Lecturer. Mr. Kenneth Adam, The Director of Television, BBC. **Guest Night,** Doctors' Ladies and Guests to be invited. At The Pump Room, Cheltenham.

5th May: **Annual Golf Meeting.** At Stinchcombe Hill Golf Club.

9th May, at 8.0 p.m.: **Annual Meeting.** Talk or Film: Mr. D. F. Robb, Assistant Secretary, The Medical Defence Union. At Gloucester.

13th June: Address: by W. H. P. Cant, Esq., M.D., F.R.C.P. Consultant Paediatrician, Birmingham. The President's Guest, at Tewkesbury.

### THE CLINICAL SOCIETY OF BATH

*Hon. Sec.:* Mr. Theo Schofield, Sunnycroft, Bloomfield Park, Bath

12th Oct.: **Clinical Meeting.** At St. Martin's Hospital.

9th Nov.: Paper by Mr. Ronald Belsey on **"The Present Scope of Thoracic Surgery".**

14th Dec.: Paper by Dr. R. Graham-Campbell on **"Incompetence of the Internal oS".** Paper by Dr. K. E. Lane on **"Emotional Illnesses".**

11th Jan.: **Clinical Meeting.**

15th Feb.: Paper by Dr. J. B. Gordon-Russell on **"The Implications of the Mental Health Act".**

15th Mar.: Paper by Professor John Bruce on **"Sense and Sensibility in the Management of Malignant Disease".**

5th Apr.: **Annual General Meeting,** followed by paper by Dr. J. T. Spare on **"Monte Carlo Rally".**

Meetings at 8.30 p.m. at Royal United Hospital unless stated otherwise. Coffee served at 8 p.m.

### TORQUAY AND DISTRICT MEDICAL SOCIETY

*Hon. Secs.:* Dr. J. B. Taylor, Corner Place, Dartmouth Road, Paignton
Dr. W. Holden, 11 Wellswood Park, Torquay

25th Oct., at 4.30 p.m.: Opening meeting.

15th Nov.: **"Chromosome Abnormalities in Medicine",** by Dr. C. O. Carter.

29th Nov.: **"Alone on the Atlantic",** by Dr. David Lewis.

13th Dec.: Symposium. I—**"Vaccination, Immunisation, Procedure and Complications",** led by Dr. D. K. MacTaggart and Dr. L. Haas. II—**"Assessment of Deafness in Children",** led by Mr. W. H. Bradbeer and Dr. L. Solomon. At Victoria Hotel, Torquay.

10th Jan.: **"Thoughts on Geriatrics",** by Dr. J. H. Simpson.

7th Feb.: **"Cardiac Surgery Today",** by Dr. D. G. Melrose.

28th Feb.: **"The Acute Abdomen",** by Mr. P. J. W. Monks.

21st Mar.: **"Congential Abnormalities from the Obstetrician's Point of View",** by Professor G. G. Lennon.

16th May: **Annual General Meeting** at Victoria Hotel, Torquay.

Meetings are held at the Torbay Hospital at 8.30 p.m. unless otherwise stated.

### B.M.A. (Barnstaple Division)

8th Nov., at 8.30 p.m.: **Clinical Meeting** at North Devon Infirmary.

1st Dec., at 6.30 p.m.: A symposium on modern trends in obstetrics. The subject will be introduced by Mr. S. Wrigley and the discussion continued by Professor Lennon. There will be a break for supper at 7.30 p.m.

10th Jan., at 8.30 p.m.: A symposium on the development of the hospital service and its relation to General Practice. This subject will be introduced by Dr. Westwater, Deputy Senior Administrative Medical Officer of the South Western Regional Hospital Board. North Devon Infirmary.

23rd Feb., at 8.30 p.m.: B.M.A. Lecture of the Year. Dr. James Cyriax will talk on Manipulative Medicine. North Devon Infirmary.

28th Mar., 8.30 p.m.: **Clinical Meeting** at Tyrell Hospital, Ilfracombe.

24th Apr., 8.30 p.m.: Annual report of Council. D. G. Lloyd-Davies Esq., F.R.C.S., will talk on Recent advances in Otorhinolaryngology. The talk will be illustrated. North Devon Infirmary.

7th June, at 8.30 p.m.: **Annual General Meeting.** Mr. Norman Capener will talk on **"Medical History in Devon: a Discussion of Ideas, Practices and Personalities".** North Devon Infirmary.

### DEVON AND EXETER MEDICO-CHIRURGICAL SOCIETY

*Hon. Sec.:* Dr. F. S. W. Brimblecombe, 15 Southernhay East, Exeter

11th Oct., at 3.30 p.m.: Lecture by Sir John Richardson on **"The Collagen Disorders".** Combined meeting with Exeter Division of the B.M.A. Exeter and South Western Medical Dinner.

1st Nov., at 3 p.m.: **Clinical Meeting.**

22nd Nov., at 8.15 p.m.: A symposium entitled **"Three Views on Asthma",** by Dr. G. F. Trobridge, Dr. Leonard Haas, Dr. Richard Hardy.

13th Dec., at 8.15 p.m.: A **Clinico-pathological Demonstration** arranged by Dr. George Stewart Smith

17th Jan., at 8.15 p.m.: A Lecture by the President on **"The Management of Diabetes"**.
7th Feb., at 8.15 p.m.: **Clinical Meeting** at the Princess Elizabeth Orthopaedic Hospital.
28th Feb., at 8.15 p.m.: A Lecture by Mr. Arthur Dickson Wright on **"Some Momentous Operations"**.
21st Mar., at 8.15 p.m.: A **Clinico-pathological Demonstration** arranged by Dr. George Stewart Smith. **Annual General Meeting.**
4th Apr., at 3 p.m.: **Clinical Meeting.**

### WESSEX RAHERE CLUB

*Hon. Sec.:* Mr. A. Daunt Bateman, 11 the Circus, Bath

A meeting of the Wessex Rahere Club, membership of which is open to all "Bart's" graduates practising in the west country, took place at the Grand Spa Hotel, Bristol, on Saturday, 20th October. Dr. E. R. Cullinan was the guest of honour.

If there are any fresh arrivals in the south-west, and they will get in touch with the Honorary Secretary, he will be only too happy to send them details.

